# Independent and joint associations of reported household chemical product use and overweight/obesity with cardiovascular disease among older adults in China: a national cross-sectional study

**DOI:** 10.3389/fnut.2026.1944461

**Published:** 2026-10-07

**Authors:** Xiaojie Pan, Jingshuang Qin, Chen Wu, Yuhan Liu, Shuchen Bai, Min Li

**Affiliations:** 1 Department of Endocrinology and Metabolism, The First Hospital of China Medical University, Shenyang, China; 2 School of Nursing, China Medical University, Shenyang, China; 3 Department of Gastrointestinal and Hernia Surgery, The First Hospital of China Medical University, Shenyang, China

**Keywords:** cardiovascular disease, CLHLS, household chemicals, older adults, overweight/obesity

## Abstract

**Background:**

Household chemicals are common indoor exposures among older adults, but their association with cardiovascular disease (CVD) and whether the association varies according to body weight status remains unclear.

**Methods:**

Data were drawn from the 2018 Chinese Longitudinal Healthy Longevity Survey. The final analysis included 12,123 adults aged 65 years or older. Reported household chemical product use frequency was classified as low or high based on the median score of eight household chemical products. Overweight/obesity was defined as BMI ≥ 24.0 kg/m^2^. CVD was defined as a composite outcome including hypertension, heart disease, or stroke. Odds ratios (ORs) and 95% confidence intervals (CIs) were estimated using binary logistic regression, with adjustment for demographic, socioeconomic, lifestyle, and health-related covariates. In addition, multiplicative and additive interactions were assessed using the product term and the relative excess risk due to interaction (RERI), attributable proportion due to interaction (AP), and synergy index (SI).

**Results:**

High-frequency household chemical product use was associated with higher odds of CVD after full adjustment (OR = 1.34, 95% CI: 1.22, 1.48, *p* < 0.001). Overweight/obesity was also associated with higher odds of CVD (OR = 2.36, 95% CI: 2.12, 2.62, *p* < 0.001). In the combined analysis, participants with both high-frequency household chemical product use and overweight/obesity had the highest odds of CVD. Additive interaction between household chemical product use and overweight/obesity was observed (RERI = 0.58, 95% CI: 0.07, 1.09; *p* = 0.013), whereas no multiplicative interaction was detected.

**Conclusion:**

Reported household chemical product use and overweight/obesity were each associated with higher odds of CVD among older adults. The combined presence of high-frequency household chemical product use and overweight/obesity was associated with the greatest odds of CVD, suggesting a potential joint contribution of environmental exposure and metabolic status to cardiovascular health.

## 1 Introduction

China is undergoing a rapid transition toward an advanced stage of population aging. In China, the population aged 65 years or older is projected to increase to approximately 366 million by 2050, accounting for about 26% of the total population (1, 2). Aging is accompanied by a gradual decline in organ function and physiological reserve, which increases susceptibility to chronic diseases (3). Overweight and obesity are also increasingly common among older adults. Global data suggest that approximately 25.3% of older adults are affected by obesity (4). In China, the prevalence of overweight among adults aged 65 years or older rose from 12.82% in 2008 to 28.45% in 2018, while the prevalence of obesity increased from 1.62 to 4.95% (5). As established risk factors for CVD, overweight and obesity may further increase the cardiovascular burden in older adults through metabolic abnormalities and related pathways (6, 7). In this context, greater attention should be given to the contribution of overweight and obesity to CVD among older Chinese adults.

Evidence suggests that a substantial proportion of CVD cases may be prevented through the management of modifiable risk factors, including smoking, unhealthy diet, physical inactivity, and air pollution (8). Among environmental factors, the association between outdoor air pollution and increased CVD risk has been well documented (9). However, older adults usually spend a substantial amount of time indoors. As a result, their indoor environmental exposures may be more continuous and more closely related to daily life (10, 11). Household chemical products represent an important and potentially modifiable source of indoor chemical exposure. Previous evidence suggests that exposure to household chemicals may be associated with respiratory diseases (12) and cognitive impairment (13) among older adults. Together, these findings indicate that household chemical exposure may have multisystem health implications. Given that the cardiovascular system is sensitive to inflammation, oxidative stress, and environmental toxic stimuli, the potential association between household chemical product use and CVD among older adults warrants further investigation.

Meanwhile, the cardiovascular associations of environmental exposures may vary according to individual health status and metabolic conditions (14, 15). Overweight/obesity may not only contribute to cardiovascular burden but also represent a metabolic state that modifies individual susceptibility to environmental stressors. Beyond excess fat accumulation, adipose tissue is recognized as an active endocrine organ, and obesity-related adipokine dysregulation may contribute to cardiometabolic alterations (16). Previous studies on outdoor air pollution have found that, compared with individuals with normal weight, those with overweight/obesity may experience stronger associations between air pollution exposure and cardiovascular outcomes (7, 17). Furthermore, a study of indoor air pollution indicated that higher concentrations of indoor pollutants, such as TVOC, were associated with increased systolic blood pressure and heart rate among individuals with higher BMI (18). These findings highlight the potential importance of considering metabolic status when examining environmental factors and cardiovascular health. However, whether similar patterns exist for reported household chemical product use remains unclear.

Based on this, our study used data from the Chinese Longitudinal Healthy Longevity Survey to examine the associations of reported household chemical product use and overweight/obesity with the odds of CVD among older adults. We further explored whether the joint association between reported household chemical product use and cardiovascular disease differed according to body weight status. These findings may help identify older adults who are more vulnerable to environment-related CVD and provide evidence for integrated prevention strategies that combine household exposure control with weight management.

## 2 Method

### 2.1 Study population

Data for this analysis were drawn from the 2018 wave of the CLHLS, a study led by the PKU Center for Healthy Aging and Development (19). The CLHLS, established in 1998 with repeated surveys every 2-3 years, adopts a multi-stage, stratified, and cluster sampling design. The survey employed a multi-stage, stratified sampling design, covering randomly selected counties across 23 of the 31 provinces in mainland China (20). Comprehensive details regarding the CLHLS research design and data collection procedures are available in prior publications (21).

The CLHLS is a nationally representative survey of older adults in China. The survey gathered comprehensive data on demographic, socioeconomic, and physical and mental health characteristics. The data of 15,874 participants were collected in the 2018 wave of the CLHLS. The initial study population included 15,771 adults aged 65 years or older. For the primary analysis, individuals with incomplete information regarding reported household chemical product use (*N* = 457), cardiovascular disease (*N* = 2004), and overweight/obesity (*N* = 1,187) were excluded. The final analysis comprised 12,123 participants (Figure 1). For covariates with missing data, complete-case analysis was used, yielding a final analytic sample of 7,087 participants for the fully adjusted model.

**Figure 1:**
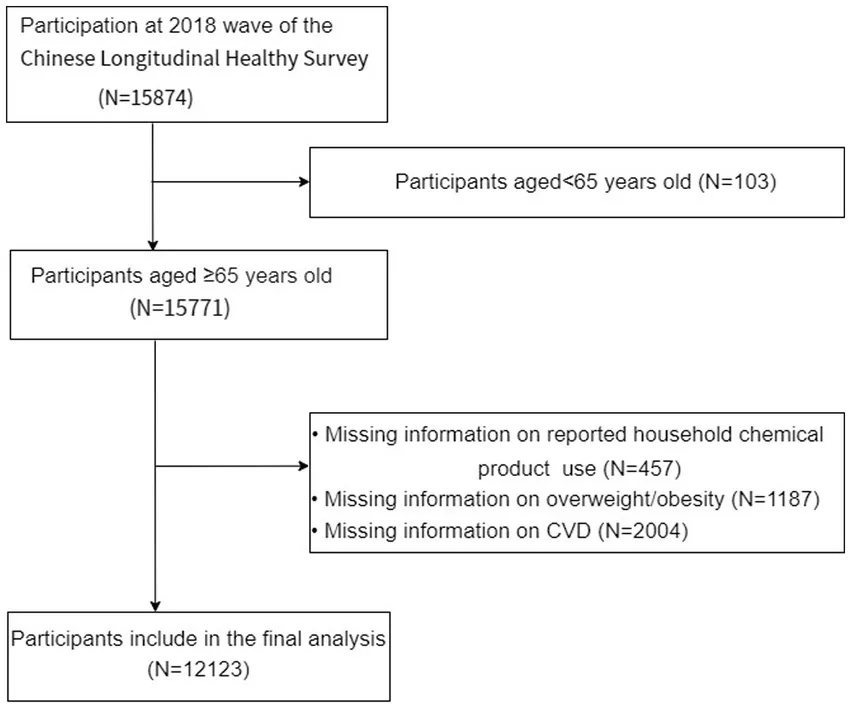
Flow chart of inclusion of participants in the study.

Ethical approval for the CLHLS was obtained from the Research Ethics Boards of Peking University and Duke University (IRB00001052-13074). Written informed consent was obtained from each participant before their inclusion in the study.

### 2.2 Reported household chemical product use

Participants were asked the following question: “Do you use these chemicals in your home?” In the CLHLS questionnaire, the household chemical products include eight items: insecticides, repellents, anti-caries agents, air fresheners, air purifiers, disinfectants, toilet cleaners, and oil removers (13). Each chemical was scored from 1–4, respectively: “rarely or never”, “seldom”, “sometimes”, and “often.” Scores for reported household chemical product use were calculated as the sum of eight items, with possible total scores ranging from 8 to 32 (12). Based on the median total score, participants were classified into low-frequency (<11) and high-frequency (≥11) household chemical product use groups.

### 2.3 Cardiovascular disease

CVD was defined as a composite outcome comprising hypertension, heart disease, and stroke (22), based on affirmative responses to three specific questions: “Do you have hypertension?” “Do you have heart disease?” and “Do you have a stroke?” If participants said “yes” to any of the aforementioned three questions, they were regarded as having CVD.

### 2.4 Overweight/Obesity

Overweight and obesity were assessed using body mass index (BMI). Height and weight were objectively measured by trained investigators and were used to calculate BMI as weight in kilograms divided by height in meters squared (kg/m^2^). According to the Chinese adult BMI classification criteria, participants were categorized as non-overweight/obesity (BMI < 24.0 kg/m^2^, including underweight and normal weight) or overweight/obesity (BMI ≥ 24.0 kg/m^2^) (23).

### 2.5 Definition of combination in reported household chemical product use and overweight/obesity

Based on the definitions of reported household chemical product use and overweight/obesity described above, participants were classified into four combined exposure groups: low-frequency household chemical product use with non-overweight status, low-frequency household chemical product use with overweight/obesity, high-frequency household chemical product use with non-overweight status, and high-frequency household chemical product use with overweight/obesity (Supplementary Table 1). In this study, the group with high-frequency household chemical product use and overweight/obesity was used as the reference group.

### 2.6 Covariates

Covariates were selected based on previous literature (12, 24, 25), theoretical considerations, and a directed acyclic graph (DAG) framework (Figure 2). The DAG was constructed to illustrate the hypothesized relationships among reported household chemical product use, overweight/obesity, CVD, and potential confounders, and to inform the selection of variables included in the multivariable models. In addition, health-related and psychosocial indicators that have been previously reported to be associated with cardiovascular health were included as additional covariates in the fully adjusted model.

**Figure 2:**
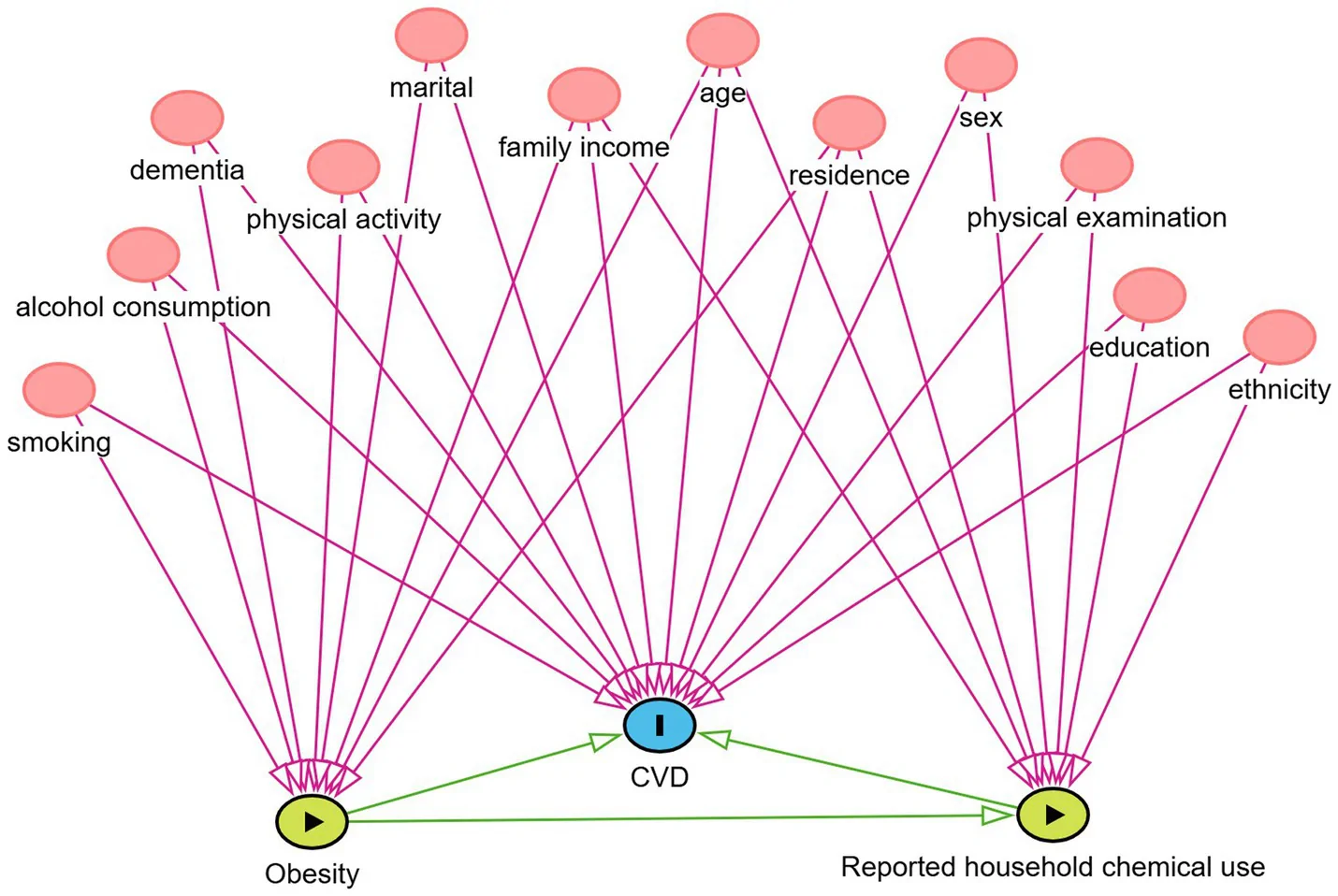
DAG illustrating the hypothesized causal assumptions underlying the association between household chemical product use, overweight/obesity, and CVD. Green nodes represent exposures; the blue node represents the outcome; pink nodes represent adjusted covariates. Green arrows represent causal paths; pink arrows represent biasing paths.

Smoking, drinking, and physical activity were categorized as binary variables according to participants’ current self-reported status. Regular physical examination was assessed using the question, “Do you undergo an annual routine physical examination?” Self-rated health was assessed using the question, “How would you rate your current health status?” Participants who responded “very good” or “good” were classified as having good self-rated health, whereas those who responded “fair,” “poor,” or “very poor” were classified as having poor self-rated health. Life satisfaction was assessed using the question, “How would you rate your life at present?” Participants who responded “very good” or “good” were classified as satisfied with life, whereas those who responded “fair,” “poor,” or “very poor” were classified as not satisfied with life.

### 2.7 Statistical analysis

The normality of continuous variables was evaluated using the Kolmogorov–Smirnov test and Q-Q plots. None of the continuous variables were normally distributed. Continuous variables are presented as medians and interquartile ranges (P25, P75), whereas categorical variables are summarized as frequencies and percentages. Comparisons between participants with and without CVD were performed using the Mann–Whitney *U* test for continuous variables and the Chi-square test for categorical variables. The complex sampling design of the CLHLS was not accounted for in the primary analyses because the present study focused on examining associations between exposures and outcomes rather than generating population-level estimates (26). Binary logistic regression was used to examine the associations of reported household chemical product use, overweight/obesity, and combined exposure groups with the odds of CVD. Four models were adjusted. Model 1 was unadjusted. Model 2 was adjusted for age and sex. Model 3 was further adjusted for ethnicity, area of residence, marital status, and educational level. Model 4 was additionally adjusted for smoking status, alcohol consumption, physical activity, physical examination, dementia, annual household income, self-rated health, and life satisfaction. To evaluate whether the findings were consistent across different cardiovascular outcomes, secondary analyses were performed using hypertension, heart disease, and stroke as separate outcomes. Additionally, a clinical CVD composite outcome excluding hypertension, defined as heart disease and/or stroke, was examined.

Multiplicative interaction between reported household chemical product use and overweight/obesity was assessed by including a product term (reported household chemical product use× overweight/obesity) in the logistic regression models, and the *p* value for interaction was reported. Additive interaction was evaluated using the RERI, AP, and SI. Stratified analyses were further performed to examine the associations of reported household chemical product use with CVD within BMI categories (non-overweight/obesity and overweight/obesity groups), and the associations of overweight/obesity with CVD within different levels of reported household chemical product use (low-frequency and high-frequency use groups). Subgroup analyses were conducted according to pre-specified demographic, socioeconomic, and health-related characteristics. Interaction tests were performed to evaluate potential heterogeneity across subgroups. In each stratified analysis, all other covariates were adjusted for, except for the stratification variable itself.

We performed several sensitivity analyses to assess the robustness of the main findings. First, missing data were addressed using multiple imputation by chained equations. Second, we excluded all participants who reported currently smoking (*n* = 1943) and drinking (*n* = 1,166) due to their established role as risk factors for CVD (27, 28). Third, we analyzed reported household chemical product use as a continuous variable. Fourth, we excluded underweight participants (BMI < 18.5 kg/m^2^) to ensure that the reference group comprised only normal-weight individuals. Fifth, overweight (BMI 24–27.9 kg/m^2^) and obesity (BMI ≥ 28 kg/m^2^) were analyzed separately. Sixth, BMI was analyzed as a continuous variable. Seventh, the reported household chemical product use score was recalculated using seven items, excluding air purifiers, as air purifier use may reflect protective behavior rather than the use of chemical products.

Data analysis was performed with IBM SPSS Statistics, version 27.0. (IBM Corp., Armonk, NY, United States) and STATA version 18.0 (Stata Corp., College Station, TX, United States) and R version 4.4.2 (R Foundation for Statistical Computing). A two-sided *p*-value of less than 0.05 was defined as statistically significant.

## 3 Results

### 3.1 Characteristics of participants

The baseline characteristics of the study participants are summarized in Table 1. The median age was 85.00 years, and 6,008 participants (49.56%) had CVD. The prevalence of individual cardiovascular conditions was 40.4% for hypertension, 16.5% for heart disease, and 10.4% for stroke. Because individual cardiovascular conditions were not mutually exclusive, their prevalences do not sum to the overall CVD prevalence. Compared with participants without CVD, those with CVD were more likely to be female, live in rural areas, have a spouse, and report no smoking, no alcohol consumption, and no regular physical activity. They were also more likely to undergo routine physical examinations and have a lower educational level. In addition, individuals with CVD also reported poorer self-rated health but higher life satisfaction.

**Table 1:** Main characteristics of the study population by CVD status.

Characteristics	Total (*N* = 12,123)	No (*N* = 6,115)	Yes (*N* = 6,008)	*p*
Reported household chemical product use				<0.001
Low	5,917 (48.81)	3,255 (53.23)	2,662 (44.31)	
High	6,206 (51.19)	2,860 (46.77)	3,346 (55.69)	
Overweight/Obesity				<0.001
No	8,409 (69.36)	4,771 (78.02)	3,638 (60.55)	
Yes	3,714 (30.64)	1,344 (21.98)	2,370 (39.45)	
Age, median (P25, P75)	85.00 (76.00, 95.00)	85.00 (76.00, 95.00)	85.00 (76.00, 95.00)	0.627
Sex, *n* (%)				0.863
Female	6,845 (56.46)	3,448 (56.39)	3,397 (56.54)	
Male	5,278 (43.54)	2,667 (43.61)	2,611 (43.46)	
Ethnicity, *n* (%)				0.011
Han	9,926 (93.99)	5,063 (94.56)	4,863 (93.39)	
Others	635 (6.01)	291 (5.44)	344 (6.61)	
Area of residence, *n* (%)				0.305
Urban	2,713 (22.38)	1,392 (22.76)	1,321 (21.99)	
Rural	9,410 (77.62)	4,723 (77.24)	4,687 (78.01)	
Marital status, *n* (%)				0.816
Have no spouse	7,021 (58.80)	3,536 (58.70)	3,485 (58.91)	
Have a spouse	4,919 (41.20)	2,488 (41.30)	2,431 (41.09)	
Smoking status, *n* (%)				0.877
No	10,180 (85.37)	5,125 (85.32)	5,055 (85.42)	
Yes	1745 (14.63)	882 (14.68)	863 (14.58)	
Drinking status, *n* (%)				0.150
No	10,169 (85.82)	5,144 (86.28)	5,025 (85.36)	
Yes	1,680 (14.18)	818 (13.72)	862 (14.64)	
Physical activity, *n* (%)				0.087
No	8,325 (70.18)	4,229 (70.90)	4,096 (69.46)	
Yes	3,537 (29.82)	1736 (29.10)	1801 (30.54)	
Physical examination, *n* (%)				<0.001
No	3,655 (30.29)	2,100 (34.52)	1,555 (25.98)	
Yes	8,413 (69.71)	3,983 (65.48)	4,430 (74.02)	
Dementia, *n* (%)				0.984
No	11,808 (98.32)	5,971 (98.32)	5,837 (98.32)	
Yes	202 (1.68)	102 (1.68)	100 (1.68)	
Education level, *n* (%)				0.935
0 year	5,237 (50.02)	2,641 (49.94)	2,596 (50.10)	
1–6 years	3,305 (31.57)	1,666 (31.51)	1,639 (31.63)	
≥7 years	1928 (18.41)	981 (18.55)	947 (18.27)	
Annual household income (yuan), *n* (%)				<0.001
<30,000	5,517 (58.44)	3,060 (61.33)	2,457 (55.20)	
≥30,000	3,923 (41.56)	1929 (38.67)	1994 (44.80)	
Self-rated health, *n* (%)				<0.001
No	5,908 (51.61)	2,529 (44.52)	3,379 (58.58)	
Yes	5,540 (48.39)	3,151 (55.48)	2,389 (41.42)	
Life satisfaction, *n* (%)				0.081
No	3,386 (29.61)	1,638 (28.86)	1748 (30.35)	
Yes	8,050 (70.39)	4,038 (71.14)	4,012 (69.65)	

Total percentages within categories may not equal 100% due to rounding.

### 3.2 Associations of reported household chemical product use and overweight/obesity with CVD

In mutually adjusted logistic regression models, both reported household chemical product use and overweight/obesity were associated with CVD. Compared with participants with low-frequency household chemical product use, those with high-frequency use had higher odds of CVD (OR = 1.34, 95% CI: 1.22, 1.48, *p* < 0.001). Similarly, overweight/obesity was associated with higher odds of CVD compared with non-overweight/obesity (OR = 2.36, 95% CI: 2.12, 2.62, *p* < 0.001). These associations remained consistent across all models (Table 2).

**Table 2:** Mutually adjusted associations of reported household chemical product use and overweight/obesity with CVD.

Variable	Reported household chemical use (high vs. low)		Overweight/Obesity (yes vs. no)	
	OR (95% CI)	*p*	OR (95% CI)	*p*
Model 1	1.39 (1.29, 1.50)	<0.001	2.28 (2.11, 2.47)	<0.001
Model 2	1.39 (1.29, 1.50)	<0.001	2.28 (2.11, 2.47)	<0.001
Model 3	1.39 (1.29, 1.51)	0.001	2.32 (2.13, 2.54)	<0.001
Model 4	1.34 (1.22, 1.48)	<0.001	2.36 (2.12, 2.62)	<0.001

OR, odds ratios; CI, confidence intervals; Ref, reference. Model 1 was unadjusted. Model 2 was adjusted for age and sex. Model 3 was adjusted for age, sex, area of residence, ethnicity, marital status, and education level. Model 4 was adjusted for age, sex, area of residence, ethnicity, marital status, smoking status, alcohol consumption, physical activity, physical examination, dementia, education, annual household income, self-rated health, and life satisfaction.

### 3.3 Association of combined analysis of reported household chemical product use and overweight/obesity with CVD

Table 3 shows the combined analysis of reported household chemical product use and overweight/obesity with CVD (Model 4). In Model 4, Participants with both high-frequency household chemical product use and overweight/obesity served as the reference group. Compared with this group, participants with low-frequency household chemical product use and non-overweight/obesity had substantially lower odds of CVD (OR = 0.31, 95% CI: 0.27, 0.36, *p* < 0.001). Those with low-frequency household chemical product use and overweight/obesity had lower odds of CVD (OR = 0.72, 95% CI: 0.60, 0.86, *p* < 0.001), as did those with high-frequency household chemical product use and non-overweight/obesity (OR = 0.41, 95% CI: 0.36–0.48, *p* < 0.001). These findings suggest that the coexistence of high-frequency household chemical product use and overweight/obesity was associated with the highest odds of CVD.

**Table 3:** Combined analysis of reported household chemical product use and overweight/obesity on CVD.

Group	Combined exposure groups	
	OR (95% CI)	*p*
Group 4	Ref	
Group 1	0.31 (0.27, 0.36)	<0.001
Group 2	0.72 (0.60, 0.86)	<0.001
Group 3	0.41 (0.36, 0.48)	<0.001

OR, odds ratios; CI, confidence intervals; Ref, reference; Group 1: low-frequency chemical product use and non-overweight/obesity; Group 2: low-frequency chemical product use and overweight/obesity; Group 3: high-frequency chemical product use and non-overweight/obesity; Group 4: high-frequency chemical product use and overweight/obesity. Adjusted for age, sex, area of residence, ethnicity, marital status, smoking status, alcohol consumption, physical activity, physical examination, dementia, education, annual household income, self-rated health, and life satisfaction.

### 3.4 Interaction between reported household chemical product use and overweight/obesity

The potential interaction between reported household chemical product use and overweight/obesity in relation to CVD was further examined on both multiplicative and additive scales. A significant additive interaction was observed between reported household chemical product use and overweight/obesity, with RERI of 0.58 (95% CI: 0.07, 1.09, *p* = 0.013), AP of 0.18 (95% CI: 0.04, 0.32, *p* = 0.007), and SI of 1.35 (95% CI: 1.03, 1.77, *p* = 0.014). However, no statistically significant multiplicative interaction was identified between reported household chemical product use and overweight/obesity (OR for interaction = 1.05, 95% CI: 0.85, 1.30; *p*-for interaction = 0.654) (Table 4).

**Table 4:** Multiplicative and additive interactions between reported household chemical product use and overweight/obesity on CVD.

Interaction type	Measure	OR (95% CI)	*p*
Multiplicative	OR for interaction	1.05 (0.85, 1.30)	0.654
Additive	RERI	0.58 (0.07, 1.09)	0.013
	AP	0.18 (0.04, 0.32)	0.007
	SI	1.35 (1.03, 1.77)	0.014

OR, odds ratios; CI, confidence intervals; RERI, relative excess risk due to interaction; AP, attributable proportion due to interaction; SI, synergy index.

### 3.5 Associations of reported household chemical product use and overweight/obesity with individual cardiovascular outcomes

To evaluate whether the observed associations differed across specific cardiovascular outcomes, we further examined hypertension, heart disease, and stroke separately, as well as a clinical CVD composite outcome excluding hypertension (heart disease and/or stroke). The associations of reported household chemical product use and overweight/obesity with individual cardiovascular outcomes were generally consistent with those observed for the primary CVD outcome (Supplementary Table 3).

### 3.6 Stratified and subgroup analyses

In stratified analyses, the association between reported household chemical product use and CVD remained significant across BMI categories (non-overweight/obesity: OR = 1.33, 95% CI: 1.18, 1.49; overweight/obesity: OR = 1.39, 95% CI: 1.17, 1.66, both *p* < 0.001). Similarly, the association between overweight/obesity and CVD remained significant across levels of reported household chemical product use (low-frequency use: OR = 2.31, 95% CI: 1.99, 2.68; high-frequency use: OR = 2.42, 95% CI: 2.08, 2.82, both *p* < 0.001) (Supplementary Table 2).

Subgroup analyses were conducted for key variables, as shown in Table 5. In the analysis of reported household chemical product use and CVD, significant interactions were observed for self-rated health (*p*-for interaction = 0.013) and life satisfaction (*p*-for interaction = 0.045). The association was weaker among participants with good self-rated health (OR = 1.34, 95% CI: 1.21, 1.49, *p* < 0.001) than among those with poor self-rated health (OR = 1.62, 95% CI: 1.46, 1.80, *p* < 0.001). A similar pattern was observed for life satisfaction, with a weaker association among participants with good life satisfaction (OR = 1.39, 95% CI: 1.28, 1.52, *p* < 0.001) than among those with poor life satisfaction (OR = 1.64, 95% CI: 1.43, 1.88, *p* < 0.001). In the analysis of overweight/obesity and CVD, the association also differed by self-rated health status (*p*-for interaction = 0.012), and was weaker among participants with good self-rated health (OR = 2.14, 95% CI: 1.91, 2.40, *p* < 0.001) than among those with poor self-rated health (OR = 2.65, 95% CI: 2.35, 2.99, *p* < 0.001).

**Table 5:** Association of reported household chemical product use and overweight/obesity with CVD among subgroups.

Variables	Reported household chemical product use			Overweight/Obesity		
	OR (95% CI)	*p*	*p*-for interaction	OR (95% CI)	*p*	*p*-for interaction
Sex			0.856			0.968
Female	1.42 (1.29, 1.56)	<0.001		2.31 (2.08, 2.57)	<0.001	
Male	1.44 (1.29, 1.61)	<0.001		2.32 (2.05, 2.61)	<0.001	
Area of residence			0.187			0.669
Urban	1.56 (1.34, 1.82)	<0.001		2.39 (2.02, 2.83)	<0.001	
Rural	1.39 (1.29, 1.51)	<0.001		2.29 (2.09, 2.51)	<0.001	
Marital status			0.890			0.549
Have no spouse	1.44 (1.31, 1.58)	<0.001		2.27 (2.04, 2.52)	<0.001	
Have a spouse	1.42 (1.27, 1.59)	<0.001		2.39 (2.10, 2.70)	<0.001	
Smoking status			0.455			0.235
No	1.44 (1.33, 1.56)	<0.001		2.27 (2.08, 2.48)	<0.001	
Yes	1.33 (1.11, 1.61)	0.003		2.61 (2.12, 3.21)	<0.001	
Drinking status			0.584			0.332
No	1.44 (1.34, 1.56)	<0.001		2.34 (2.14, 2.55)	<0.001	
Yes	1.36 (1.12, 1.65)	<0.001		2.09 (1.70, 2.58)	<0.001	
Physical activity			0.251			0.726
No	1.39 (1.27, 1.51)	<0.001		2.33 (2.12, 2.57)	<0.001	
Yes	1.52 (1.33, 1.74)	<0.001		2.26 (1.95, 2.62)	<0.001	
Physical examination			0.074			0.667
No	1.30 (1.14, 1.48)	<0.001		2.31 (1.98, 2.70)	<0.001	
Yes	1.50 (1.37, 1.63)	<0.001		2.22 (2.03, 2.44)	<0.001	
Dementia			0.093			0.609
No	1.44 (1.34, 1.54)	<0.001		2.33 (2.15, 2.53)	<0.001	
Yes	0.89 (0.51, 1.55)	0.679		1.93 (0.95, 3.94)	0.069	
Education level			0.140			0.137
0 year	1.51 (1.36, 1.69)	<0.001		2.30 (2.04, 2.60)	<0.001	
1–6 years	1.28 (1.12, 1.47)	<0.001		2.61 (2.12, 3.21)	<0.001	
≥7 years	1.53 (1.28, 1.83)	<0.001		2.03 (1.66, 2.48)	<0.001	
Annual household income (yuan)			0.524			0.100
<30,000	1.29 (1.15, 1.43)	<0.001		2.14 (1.90, 2.41)	<0.001	
≥30,000	1.36 (1.20, 1.54)	<0.001		2.49 (2.17, 2.87)	<0.001	
Self-rated health			0.013			0.012
Poor	1.62 (1.46, 1.80)	<0.001		2.65 (2.35, 2.99)	<0.001	
Good	1.34 (1.21, 1.49)	<0.001		2.14 (1.91, 2.40)	<0.001	
Life satisfaction			0.045			0.112
Poor	1.64 (1.43, 1.88)	<0.001		2.54 (2.17, 1.97)	<0.001	
Good	1.39 (1.28, 1.52)	<0.001		2.19 (1.99, 2.41)	<0.001	

OR, odds ratios; CI, confidence intervals.

### 3.7 Sensitivity analyses

Several sensitivity analyses were conducted to examine whether the main findings were robust. The associations remained consistent across all sensitivity analyses. First, after multiple imputation for missing covariates, the results were concordant with those from the complete-case analysis (all *p* < 0.001). Second, after excluding current smokers and drinkers, the observed associations remained statistically significant (all *p* < 0.001) (Supplementary Tables 4–6). Third, when reported household chemical product use was analyzed as a continuous variable, similar results were obtained (all *p* < 0.001) (Supplementary Table 7). Fourth, after excluding underweight participants (BMI < 18.5 kg/m^2^), the associations remained robust (high-frequency reported household chemical product use: OR = 1.33, 95% CI: 1.20, 1.48; overweight/obesity: OR = 2.17, 95% CI: 1.94, 2.42; all *p* < 0.001) (Supplementary Table 8). Fifth, when overweight (BMI 24–27.9 kg/m^2^) and obesity (BMI ≥ 28 kg/m^2^) were analyzed separately, both were significantly associated with CVD, with a stronger association observed for obesity than overweight (overweight: OR = 1.98, 95% CI: 1.75, 2.23; obesity: OR = 2.99, 95% CI: 2.44, 3.66; both *p* < 0.001). Sixth, when BMI was analyzed as a continuous variable, each 1 kg/m^2^ increment in BMI was associated with higher odds of CVD (OR = 1.13, 95% CI: 1.12, 1.15, *p* < 0.001) (Supplementary Table 9). Seventh, when the household chemical product use score was recalculated using seven items (excluding air purifiers), the associations remained consistent with the main analysis (all *p* < 0.001) (Supplementary Table 10).

## 4 Discussion

This study showed that both reported household chemical product use and overweight/obesity were associated with higher odds of CVD among older adults. Further joint analyses showed that among participants with high-frequency reported household chemical use, those with overweight/obesity had higher odds of CVD than those without overweight/obesity. Participants with both high-frequency reported household chemical product use and overweight/obesity had the highest odds of CVD. Furthermore, a significant additive interaction was observed between reported household chemical product use and overweight/obesity (RERI = 0.58, *p* = 0.013), although the multiplicative interaction was not significant (*p* = 0.654). The associations were consistent across CVD subtypes (hypertension, heart disease, and stroke). These findings suggest that the association between reported household chemical product use and CVD may differ according to body weight status. In addition, stratified analyses suggested that, in the association of reported household chemical product use with CVD, participants with better self-rated health and higher life satisfaction had relatively lower odds of CVD. Similarly, in the association between overweight/obesity and CVD, participants with better self-rated health also showed lower odds of CVD.

Previous studies have suggested that reported household chemical product use may be associated with alterations in multiple organ systems, including respiratory health among children (29) and older adults (12), and may also be related to cardiovascular health (30). The present study adds evidence from older Chinese adults by showing that reported household chemical product use was associated with higher odds of CVD. Potential mechanisms proposed in previous studies include oxidative stress, systemic inflammation, endothelial dysfunction, and metabolic disturbances associated with certain chemical components (31–33). Older adults may be particularly susceptible to these associations because of reduced physiological reserve and a higher prevalence of cardiometabolic conditions. In addition to reported household chemical product use, individual metabolic characteristics may also be relevant to cardiovascular health. Overweight and obesity are well-established risk factors for CVD, as consistently demonstrated in both US and Chinese populations (6, 34). Our findings support previous evidence that overweight/obesity is positively associated with the odds of CVD in older Chinese adults. Biologically, adipose tissue is increasingly recognized as an active endocrine organ rather than merely an energy storage site. Excess adiposity can lead to adipose tissue dysfunction, characterized by altered adipokine secretion, including reduced adiponectin levels and increased production of pro-inflammatory mediators (16). These alterations may contribute to chronic low-grade inflammation, metabolic dysfunction, and endothelial impairment, thereby promoting atherosclerosis and hypertension and increasing susceptibility to CVD (35–39). Notably, reported household chemical product use and overweight/obesity may share common pathophysiological pathways. Therefore, this study further examined their joint association with CVD risk among older Chinese adults.

The combined analysis showed that participants with both high-frequency reported household chemical product use and overweight/obesity had the highest odds of CVD. This finding extends previous evidence by suggesting that environmental-related behaviors and metabolic conditions may jointly contribute to cardiovascular burden among older adults. Previous studies have reported that the cardiovascular associations of environmental pollutants may vary according to individual metabolic characteristics. For example, studies of outdoor air pollution have observed stronger associations between air pollution exposure and cardiovascular outcomes among individuals with overweight/obesity compared with those with normal weight (17). Similarly, a study of indoor air pollution among office workers found that higher concentrations of indoor pollutants, such as TVOC, were associated with increased systolic blood pressure and heart rate among individuals with higher BMI (18). Together, these findings support the importance of considering metabolic status when investigating the cardiovascular implications of environmental factors. Our study further extends this evidence by focusing on reported household chemical use, an indoor environmental factor commonly encountered in daily life.

Several biological pathways may help explain the observed joint association between reported household chemical product use and overweight/obesity. Overweight/obesity is characterized by adipose tissue dysfunction, which may involve chronic low-grade inflammation, altered adipokine secretion, and metabolic disturbances (40–42). Meanwhile, certain chemical components that may be present in household products, such as volatile organic compounds and phthalates, have been associated with oxidative stress, inflammatory responses, and metabolic disruption (43). Therefore, the coexistence of metabolic abnormalities and reported household chemical product use may reflect overlapping physiological challenges that are relevant to cardiovascular health. This interpretation is consistent with emerging multi-organ and dynamic health trajectory frameworks, which emphasize that chronic health outcomes may arise from interconnected changes across multiple biological systems rather than isolated pathways (44). Such a framework provides a broader perspective for understanding the potential interplay between environment-related behaviors, metabolic conditions, and cardiovascular health. In line with this perspective, a significant additive interaction was observed between reported household chemical product use and overweight/obesity. Together, these findings indicate that their combined association with CVD exceeded the expected association based on their individual associations.

These findings have certain public health implications. Household chemicals are widely used in daily life, and complete avoidance of exposure may not be realistic for older adults, especially for those who spend a long time indoors. The present study found that maintaining a healthier body weight was associated with lower odds of CVD when reported household chemical product use persists. Therefore, CVD prevention among older adults may consider both environmental exposure control and weight management. Community health education may encourage older adults to reduce the unnecessary use of highly volatile products, such as air fresheners, disinfectants, and insecticides, improve indoor ventilation, and incorporate a healthy diet, regular physical activity, and weight management into daily health care. For older adults with both reported household chemical product use and overweight/obesity, targeted risk identification and intervention should be strengthened.

Several limitations of this study should be acknowledged. First, the cross-sectional design precludes conclusions regarding causality, and longitudinal studies are needed to establish temporal relationships and further clarify these associations. Second, household chemical product use, CVD status, and several covariates were based on self-reported information, which may be subject to recall bias or social desirability bias. Future studies incorporating objective measurements of household chemical-related exposures and clinical outcomes are needed to improve measurement accuracy. Third, although the CLHLS employed a multistage, stratified, and cluster sampling design, sampling weights were not applied in the present study because the primary aim was to examine associations rather than estimate national prevalence rates (26). Fourth, the reported household chemical product use score combined eight heterogeneous items with potentially different chemical compositions and biological relevance. The equal-weighting approach was applied because no validated weighting method is currently available. Future studies with detailed exposure characterization and validated scoring approaches are warranted. Finally, participants were exclusively older adults from mainland China; therefore, the generalizability of these findings to younger populations or other geographic settings requires further investigation. Finally, the proposed mechanisms linking reported household chemical use and overweight/obesity to CVD—including inflammatory, oxidative, endothelial, and adipokine-related pathways—are biologically plausible but were not directly measured in this study. Future research with objective biomarkers is needed to confirm these pathways.

## 5 Conclusion

In conclusion, this national cross-sectional study found that high-frequency household chemical product use and overweight/obesity were independently associated with higher odds of CVD among older adults in China. Participants with both high-frequency household chemical product use and overweight/obesity showed the highest odds of CVD, suggesting that the association between reported household chemical product use and CVD may vary according to body weight status. These findings highlight the potential importance of considering household environmental exposures and weight status in cardiovascular health research and prevention strategies for older adults. Future longitudinal studies incorporating objective exposure assessments are needed to confirm these associations and further explore potential mechanisms.

## Data Availability

The datasets utilized for this study are available on the National Archive of Computerized Data on Aging (NACDA), access link: https://www.icpsr.umich.edu/icpsrweb/NACDA/series/487.
