# Supplementary material for: Independent and joint associations of reported household chemical product use and overweight/obesity with cardiovascular disease among older adults in China: a national cross-sectional study
Source: Front Nutr. 2026 Oct 7;13:1944461. doi: 10.3389/fnut.2026.1944461 (PMC13683573; doi:10.3389/fnut.2026.1944461)
Supplement: Supplementary file 1 [file Data_Sheet_1.PDF]

Supplementary Table 1. Definition of the four combined exposure groups

Group	Total n (%)	Reported household chemical product use	Overweight/obesity
Group 1	4253 (35.08)	Low - frequency	Non-overweight/obesity
Group 2	1664 (13.73)	Low - frequency	overweight/obesity
Group 3	4156 (34.28)	High - frequency	Non-overweight/obesity
Group 4	2050 (16.91)	High - frequency	overweight/obesity

Supplementary Table 2. Stratified associations of reported household chemical product use and overweight/obesity with CVD

Stratification	OR (95%CI)	P
**Reported household chemical product use and CVD**		
Non-overweight/obesity	1.33 (1.18, 1.49)	<0.001
Overweight/obesity	1.39 (1.17, 1.66)	<0.001
**Overweight/obesity and CVD**		
Low-frequency chemical use	2.31 (1.99, 2.68)	<0.001
High-frequency chemical use	2.42 (2.08, 2.82)	<0.001

Abbreviation: OR: Odds ratios; CI: Confidence intervals; CVD: cardiovascular disease.

Adjusted for age, sex, area of residence, ethnicity, marital status, smoking status, alcohol consumption, physical activity, physical examination, dementia, education, annual household income, self-rated health, and life satisfaction.

Supplementary Table 3 Associations of reported household chemical product use and overweight/obesity with individual cardiovascul ar outcomes

Outcome	Reported household chemical product use (High vs Low)		Overweight/obesity (Yes vs No)	
	OR (95%CI)	P	OR (95%CI)	P
Hypertension	1.38 (1,25, 1.53)	<0.001	2.38 (2.14, 2.65)	<0.001
Heart disease	1.36 (1.19, 1.55)	<0.001	1.85 (1.62, 2.11)	<0.001
Stroke	1.31 (1.11, 1.54)	0.001	1.71 (1.45, 2.02)	<0.001
CVD excluding hypertension	1.34 (1.20, 1.51)	<0.001	1.81 (1.61, 2.05)	<0.001

Abbreviation: OR: Odds ratios; CI: Confidence intervals; CVD: cardiovascular disease.

Adjusted for age, sex, area of residence, ethnicity, marital status, smoking status, alcohol consumption, physical activity, physical examination, dementia, education, annual household income, self-rated health, and life satisfaction.

Supplementary Table 4 Sensitivity analyses for the reported household chemical product use with CVD

	After multiple imputation		Excluding current smokers and drinkers	
	OR (95%CI)	P	OR (95%CI)	P
Model 1	1.43 (1.33, 1.54)	<0.001	1.45 (1.34, 1.58)	<0.001
Model 2	1.43 (1.33, 1.54)	<0.001	1.45 (1.34, 1.58)	<0.001
Model 3	1.43 (1.33, 1.53)	<0.001	1.45 (1.33, 1.59)	<0.001
Model 4	1.39 (1.29, 1.49)	<0.001	1.39 (1.25, 1.55)	<0.001

Abbreviation: OR: Odds ratios; CI: Confidence intervals; CVD: cardiovascular disease.

Model 1 was unadjusted.

Model 2 was adjusted for age, sex.

Model 3 was adjusted for age, sex, area of residence, ethnicity, marital status, and education level.

Model 4 was adjusted for age, sex, area of residence, ethnicity, marital status, smoking status, alcohol consumption, physical activity, physical examination, dementia, education, annual household income, self-rated health and life satisfaction.

Supplementary Table 5 Sensitivity analyses for the Overweight/obesity with CVD

	After multiple imputation		Excluding current smokers and drinkers	
	OR (95%CI)	P	OR (95%CI)	P
Model 1	2.31 (2.14, 2.50)	<0.001	2.26 (2.06, 2.48)	<0.001
Model 2	2.31 (2.14, 2.50)	<0.001	2.26 (2.06, 2.48)	<0.001
Model 3	2.31 (2.14, 2.51)	<0.001	2.31 (2.09, 2.56)	<0.001
Model 4	2.32 (2.13, 2.51)	<0.001	2.34 (2.07, 2.64)	<0.001

Abbreviation: OR: Odds ratios; CI: Confidence intervals; CVD: cardiovascular disease.

Model 1 was unadjusted.

Model 2 was adjusted for age, sex.

Model 3 was adjusted for age, sex, area of residence, ethnicity, marital status, and education level.

Model 4 was adjusted for age, sex, area of residence, ethnicity, marital status, smoking status, alcohol consumption, physical activity, physical examination, dementia, education, annual household income, self-rated health and life satisfaction.

Supplementary Table 6 Sensitivity analyses for the combined analysis of reported household chemical product use and overweight/obesity on CVD

	After multiple imputation		Excluding current smokers and drinkers	
	OR (95%CI)	P	OR (95%CI)	P
Group 4	Ref		Ref	
Group 1	0.32 (0.28, 0.36)	< 0.001	0.32 (0.27, 0.38)	< 0.001
Group 2	0.70 (0.61, 0.80)	< 0.001	0.74 (0.60, 0.90)	< 0.001
Group 3	0.42 (0.37, 0.47)	< 0.001	0.43 (0.36, 0.51)	< 0.001

Abbreviation: OR: Odds ratios; CI: Confidence intervals; CVD: cardiovascular disease; Ref: reference. Group 1: low-frequency chemical product use and non-overweight/obesity; Group 2: low-frequency chemical product use and overweight/obesity; Group 3: high-frequency chemical product use and non-overweight/obesity; Group 4: high-frequency chemical product use and overweight/obesity.

Model 4 was adjusted for age, sex, area of residence, ethnicity, marital status, smoking status, alcohol consumption, physical activity, physical examination, dementia, education, annual household income, self-rated health and life satisfaction.

Supplementary Table 7 Association of reported household chemical product use as continuous variable with CVD

	Reported household chemical product use	
Model	OR (95%CI)	P
Model 1	1.06 (1.04, 1.07)	<0.001
Model 2	1.06 (1.04, 1.07)	<0.001
Model 3	1.05 (1.04, 1.06)	<0.001
Model 4	1.05 (1.04, 1.07)	<0.001

Abbreviation: OR: Odds ratios; CI: Confidence intervals; CVD: cardiovascular disease.

Model 1 was unadjusted.

Model 2 was adjusted for age, sex.

Model 3 was adjusted for age, sex, area of residence, ethnicity, marital status, and education level.

Model 4 was adjusted for age, sex, area of residence, ethnicity, marital status, smoking status, alcohol consumption, physical activity, physical examination, dementia, education, annual household income, self-rated health and life satisfaction.

Supplementary Table 8. Sensitivity analysis excluding underweight participants

Analysis	OR (95%CI)	P
Reported household chemical product use (High vs Low)	1.33 (1.20, 1.48)	<0.001
Overweight/Obesity (Yes vs No)	2.17 (1.94, 2.42)	<0.001
Joint effect (Group 4 as reference)		
Group 1	0.35 (0.30, 0.41)	<0.001
Group 2	0.72 (0.60, 0.86)	<0.001
Group 3	0.44 (0.38, 0.52)	<0.001

Abbreviation: OR, odds ratio; CI, confidence interval; Group 1: low-frequency chemical product use and non-overweight/obesity; Group 2: low-frequency chemical product use and overweight/obesity; Group 3: high-frequency chemical product use and non-overweight/obesity; Group 4: high-frequency chemical product use and overweight/obesity.

Adjusted for age, sex, area of residence, ethnicity, marital status, smoking status, alcohol consumption, physical activity, physical examination, dementia, education, annual household income, self-rated health, and life satisfaction.

Supplementary Table 9 Sensitivity analyses for BMI classification and CVD

Analysis	OR (95%CI)	P
Normal weight	Ref	
Overweight	1.98 (1.75, 2.23)	<0.001
Obesity	2.99 (2.44, 3.66)	<0.001
BMI as continuous variable	1.13 (1.12, 1.15)	<0.001

Abbreviation: OR: Odds ratios; CI: Confidence intervals; CVD: cardiovascular disease; BMI: Body Mass Index; Ref: reference.

Adjusted for age, sex, area of residence, ethnicity, marital status, smoking status, alcohol consumption, physical activity, physical examination, dementia, education, annual household income, self-rated health, and life satisfaction.

Supplementary Table 10 Sensitivity analysis excluding air purifiers from the reported household chemical product use score

Analysis	OR (95%CI)	P
Reported household chemical product use (High vs Low)	1.38 (1.25, 1.51)	<0.001
Joint effect (Group 4 as reference)		
Group 1	0.31 (0.27, 0.36)	<0.001
Group 2	0.72 (0.60, 0.86)	<0.001
Group 3	0.41 (0.36, 0.48)	<0.001

Abbreviation: OR, odds ratio; CI, confidence interval; Group 1: low-frequency chemical product use and non-overweight/obesity; Group 2: low-frequency chemical product use and overweight/obesity; Group 3: high-frequency chemical product use and non-overweight/obesity; Group 4: high-frequency chemical product use and overweight/obesity.

Adjusted for age, sex, area of residence, ethnicity, marital status, smoking status, alcohol consumption, physical activity, physical examination,

dementia, education, annual household income, self-rated health, and life satisfaction.
